# Genetic, cellular, and connectomic characterization of the brain regions commonly plagued by glioma

**DOI:** 10.1093/brain/awaa277

**Published:** 2020-12-04

**Authors:** Ayan S Mandal, Rafael Romero-Garcia, Michael G Hart, John Suckling

**Affiliations:** 1 Brain Mapping Unit, Department of Psychiatry, University of Cambridge, Cambridge, UK; 2 Academic Division of Neurosurgery, Department of Clinical Neurosciences, University of Cambridge, Cambridge, UK

**Keywords:** neuro-oncology, gliomagenesis, connectomics, imaging-transcriptomics

## Abstract

For decades, it has been known that gliomas follow a non-random spatial distribution, appearing more often in some brain regions (e.g. the insula) compared to others (e.g. the occipital lobe). A better understanding of the localization patterns of gliomas could provide clues to the origins of these types of tumours, and consequently inform treatment targets. Following hypotheses derived from prior research into neuropsychiatric disease and cancer, gliomas may be expected to localize to brain regions characterized by functional hubness, stem-like cells, and transcription of genetic drivers of gliomagenesis. We combined neuroimaging data from 335 adult patients with high- and low-grade glioma to form a replicable tumour frequency map. Using this map, we demonstrated that glioma frequency is elevated in association cortex and correlated with multiple graph-theoretical metrics of high functional connectedness. Brain regions populated with putative cells of origin for glioma, neural stem cells and oligodendrocyte precursor cells, exhibited a high glioma frequency. Leveraging a human brain atlas of post-mortem gene expression, we found that gliomas were localized to brain regions enriched with expression of genes associated with chromatin organization and synaptic signalling. A set of glioma proto-oncogenes was enriched among the transcriptomic correlates of glioma distribution. Finally, a regression model incorporating connectomic, cellular, and genetic factors explained 58% of the variance in glioma frequency. These results add to previous literature reporting the vulnerability of hub regions to neurological disease, as well as provide support for cancer stem cell theories of glioma. Our findings illustrate how factors of diverse scale, from genetic to connectomic, can independently influence the anatomic localization of brain dysfunction.

## Teaser

Using neuroimaging and genetic methods, Mandal *et al.* show that gliomas localize to brain regions characterized by functional hubness, stem-like cells, and transcription of genes involved in tumour development. The results illustrate how factors of diverse scale can collectively determine the location of brain dysfunction.

## Introduction

Tumour location represents one of the most important prognostic factors for patients suffering from primary brain cancers (Jeremic *et al.*, 1994; Sagberg *et al.*, 2019), yet little is known about the mechanisms that determine the spatial distribution of gliomas across the brain.

The importance of glioma location for diagnosis and treatment has been recognized since Percival Bailey and Harvey Cushing’s seminal classification of brain tumours in the early 20th century (Bailey and Cushing, 1926). Whilst brain imaging, primarily MRI, plays an important and routine role in diagnosis and treatment of brain tumours, there has been little quantitative mapping of their distribution at a population level. Comprehensive modelling of the key factors involved in determining why gliomas might be heterogeneously distributed across the brain could shed light on the origins of these tumours, and consequently inform treatment targets. Three general hypotheses for the spatial distribution of gliomas include a ‘connectomic hypothesis’, a ‘cellular hypothesis’, and a ‘genetic hypothesis’, and each is now considered in turn.

The connectomic hypothesis posits that highly connected brain regions, known as hubs, are especially vulnerable to disorders, such as oncogenesis, due to the metabolic costliness of maintaining many connections (Bullmore and Sporns, 2012; Crossley *et al.*, 2014). According to network neuroscience theory, efficient communication across the brain is crucially dependent upon hubs that facilitate information transfer both within their own communities and between diverse subsystems (van den Heuvel and Sporns, 2013). Brain hubs are believed to be ‘costly’ due to the metabolic demand of maintaining many connections (Bullmore and Sporns, 2012), a factor that makes these regions vulnerable to disease (Seeley *et al.*, 2009; Crossley *et al.*, 2014; Warren *et al.*, 2014; Aerts *et al.*, 2016). Long distance axonal connections for instance, are physiologically expensive to maintain since proteins in the neuron’s presynaptic terminal must be produced in the nucleus, and thus travel the full distance of the axon to reach their target. This factor contributes to the vulnerability of upper motor neurons to degeneration (Nijssen *et al.*, 2017). In a similar way, long distance connections important for the construction of large-scale cortical networks also pose a challenge for glial cells (in particular, oligodendrocytes) to support the requisite axonal tracts (Buzsáki *et al.*, 2004). Furthermore, brain hubs also receive many connections, and therefore are populated with many synapses that impose metabolic demand upon supporting astrocytes (Magistretti and Allaman, 2015). Metabolic demands on the glial cells of hub regions could contribute to elevated cell turnover, enhancing the likelihood of a cell acquiring an oncogenic mutation during mitosis (Wodarz, 2007). Metabolic stress could also contribute to oncogenesis via enhanced production of mutagenic reactive oxygen species (Rinaldi *et al.*, 2016). For these reasons, one may expect gliomas to localize to hubs of the brain’s connectome.

With their shared dedifferentiated and proliferative nature, the commonalities between stem cells and cancer cells have not gone unnoticed among cancer biologists. These commonalities form the basis of the stem cell hypothesis of cancer, which maintains that cancers tend to originate from normal stem and stem-like cells in the body (Sanai *et al.*, 2005; Visvader, 2011; Jiang and Uhrbom, 2012). When applied to adult glioma, this hypothesis points to two clear suspects as possible cells of origin: neural stem cells (NSCs) and oligodendrocyte precursor cells (OPCs; Sanai *et al.*, 2005; Jiang and Uhrbom, 2012). Neither are randomly distributed throughout the brain, and therefore their specific localization patterns have been hypothesized to play a role in determining the non-random distribution of gliomas (Zlatescu *et al.*, 2001; Mueller *et al.*, 2002). The notion that neural stem cells exist and continue to proliferate in the adult human brain is relatively new and historically controversial, but a consensus has arisen that they can be found in at least two locations: the dentate gyrus of the hippocampus, and the subventricular zone (Sanai *et al.*, 2005; Ma *et al.*, 2009). Rodent work has demonstrated that oligodendrocyte precursor cells are widely distributed throughout the mammalian brain (Hughes *et al.*, 2013). The patterning of oligodendrocyte precursor cells in the adult human brain is unclear, but could be estimated by utilizing brain-wide maps of gene expression patterns (Hawrylycz *et al.*, 2012; Seidlitz *et al.*, 2020).

Adult gliomagenesis is the result of glial cells acquiring a series of somatic mutations that trigger uncontrollable cell proliferation (Reifenberger *et al.*, 2017; Molinaro *et al.*, 2019). Recent research has demonstrated that tumour location is influenced by the genetic aberrations that guide tumour development (Zlatescu *et al.*, 2001; Tejada Neyra *et al.*, 2018). Gliomas may be expected to localize to brain regions where the genetic risk factors for the disease are normatively expressed. Furthermore, consequential to the connectomic and cellular hypotheses, it may be expected that brain regions frequented by glioma are enriched with the expression of genes associated with cell proliferation or metabolically intensive processes required for long distance neuronal signalling.

In this study, we tested these three hypotheses by examining the connectomic, cellular, and genetic correlates of brain regions commonly plagued by glioma. We began by deriving a replicable tumour frequency map from neuroimaging data of 335 adult patients with high- and low-grade glioma. Using this map, we compared glioma distributions across canonical subnetworks and correlated them with hub measures calculated from averaged functional connectivity data from a large number of healthy individuals. Then, we determined if glioma frequency was elevated among brain regions expected to be enriched with neural stem cells and OPCs. Next, we conducted a transcriptomic analysis to find genes with spatial expression patterns that followed the observed glioma distribution. Finally, we combined all these factors of glioma distribution into a single regression model to explore the putative inter-relationships of predictors of glioma frequency.

## Materials and methods

### Tumour frequency map

Neuroimaging data of patients with low and high grade gliomas were accessed from the Multimodal Brain Tumor Image Segmentation Challenge 2019 (BraTS: http://braintumorsegmentation.org; Menze *et al.*, 2015; Bakas *et al.*, 2017, 2018). T_1_-weighted contrast-enhanced scans from 259 patients with high-grade glioma and 76 patients with low-grade glioma were segmented by board-certified neuroradiologists, denoting voxels that constituted gadolinium enhancing tumour, non-enhancing core, and peritumoural oedema (Bakas *et al.*, 2017). Segmentation was informed by multimodal imaging, including T_1_-weighted, post-contrast T_1_-weighted, T_2_-weighted, and T_2_ fluid inversion attenuated recovery (T_2_-FLAIR) scans. Scans were acquired at 19 different institutions (https://www.med.upenn.edu/cbica/brats2019/people.html) with different sequences and protocols. These data were preprocessed through the same pipeline, undergoing linear registration to a common template (SRI24; Rohlfing *et al.*, 2010), resampling at 1 mm^3^ isotropic resolution, and removing non-brain tissues from the image (Bakas *et al.*, 2018).

The minimally preprocessed images were downloaded from the Center for Biomedical Image Computing and Analytics Image Processing Portal (CBICA IPP). Images from each patient were non-linearly warped to a common template (Rohlfing *et al.*, 2010) using Advanced Normalization Tools software (ANTS; Avants *et al.*, 2009), with cost-function masking of abnormal brain tissue. The registered masks comprising the gadolinium enhancing tumour and non-enhancing core were taken to represent (and hereafter will be referred to as) the tumour mask.

**Figure 1:**
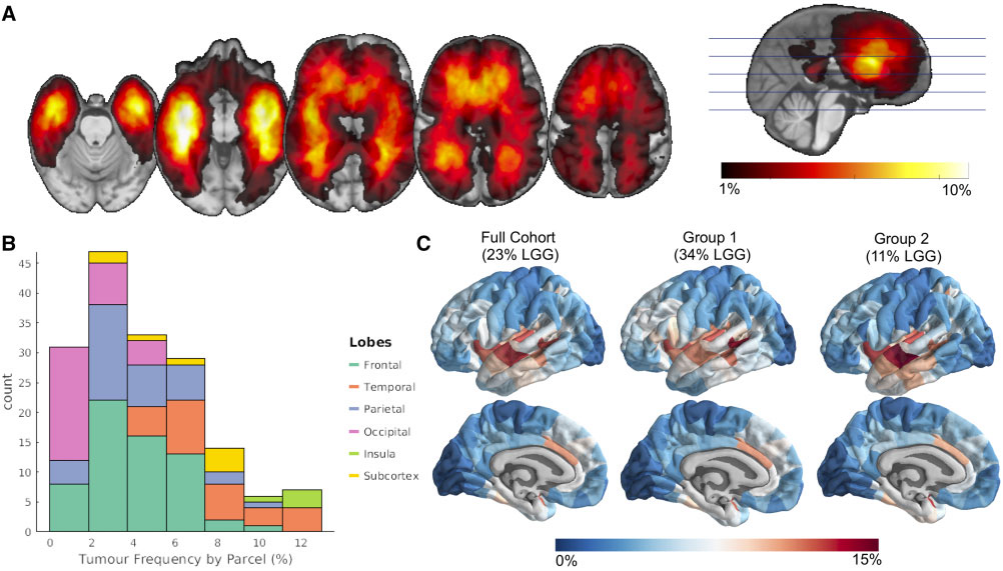
**Non-random spatial distribution of gliomas.** (**A**) Tumour frequency map derived from lesion masks from 335 patients with high and low grade glioma (LGG). (**B**) Glioma frequency by common anatomic subdivisions. (**C**) Glioma frequency represented at a parcel-level. Internal replicability of glioma frequency tested by constructing two independent maps from even splits of the cohort, where the first comprised ∼34% low grade gliomas and the other of ∼11% low grade gliomas.

Tumour masks were concatenated across all 335 patients to create a tumour frequency map, where the value at each voxel denotes the percentage of tumours of the sample that overlapped with that voxel (Fig. 1A). Smoothing with a 2 mm full-width at half-maximum (FWHM) Gaussian kernel was applied to the map. An unsmoothed version of this map is shown in Supplementary Fig. 1. To match genetic data for which most of the samples come from one hemisphere (Hawrylycz *et al.*, 2012), we mirrored the tumour frequency map to the left hemisphere for the following analyses. Given the large sample size and concordance with other studies (Duffau and Capelle, 2004; Larjavaara *et al.*, 2007), this tumour frequency map was interpreted as representing general glioma spatial distribution.

### Tumour frequency parcellation

To quantify tumour frequency by common anatomic subdivisions, we applied to the tumour frequency map an in-house 334 region (with 167 left hemisphere regions) parcellation covering 16 subcortical and 318 neocortical areas. This symmetric parcellation was created by applying a back-tracking algorithm that restricts the parcel size to 500 mm^2^ with the Desikan-Killany atlas boundaries as starting points (Romero-Garcia *et al.*, 2012). Although this parcellation was grey matter based, parcels were extended 4 mm into the white matter to capture tumour frequency in adjacent white matter regions. Tumour frequency for a parcel was calculated by averaging the voxel value (representing percent tumour overlap) of the mirrored tumour frequency map within each left hemisphere parcel.

### Internal replicability

The internal replicability of our tumour frequency map was tested by correlating tumour frequency maps derived from randomly assigned, non-overlapping cohorts of 168 and 167 patients (Groups 1 and 2).

Simultaneously, we tested the generalizability of our results to groups constituted of differing proportions of low grade versus high grade gliomas. The first group (Group 1) had a 50% higher proportion of low grade gliomas (∼34%) as the full cohort (∼23%), whereas the second group (Group 2) was constituted of a 50% lower proportion of low grade glioma patients (∼11%). These tumour frequency maps were constructed with the same processing as that with the full sample (smoothing, mirroring to the left hemisphere, and parcellation). A 95% confidence interval (CI) for the inter-parcel correlation between Groups 1 and 2 was determined by constructing a distribution of 100 correlation coefficients where different patients were selected for each group.

### Statistical inference of brain map correspondence

Several analyses in this study involved investigating the spatial correspondence between different imaging-derived measures. In general, this was accomplished by calculating the measures at each parcel in the common parcellation scheme, then correlating these measures across parcels for hypothesis testing. However, since the spatial resolution (and thus the number of parcels) of any parcellation scheme is essentially arbitrary, the actual degrees of freedom cannot be estimated. This is aggravated by the spatial autocorrelation of measures among neighbouring parcels that violates the assumption of independent observations. We addressed this concern by applying a spatial randomization scheme to our relatively coarse-grained, 167-region parcellation, to conduct non-parametric hypothesis testing of brain map correspondence. To mitigate the effects of randomization on spatial autocorrelation, we used the ‘spin test’, which has been used in past studies to address this problem (Alexander-Bloch *et al.*, 2013, 2018; Vandekar *et al.*, 2015; Váša *et al.*, 2018). The spin test procedure is described in more detail in the Supplementary material. In general, it involves comparing the observed inter-parcel correlation between maps of two measures with a distribution of correlations calculated after one of these maps has been spatially permuted in a way that preserves contiguity among parcels.

### Comparison of glioma frequency across canonical subnetworks

One question of interest was whether gliomas localized to particular brain subnetworks. Seven canonical subnetworks of the brain (https://surfer.nmr.mgh.harvard.edu/fswiki/CorticalParcellation_Yeo2011; Yeo *et al.*, 2011) were mapped onto the tumour frequency map. These networks were originally defined using a clustering approach applied to resting state functional MRI data from 1000 individuals (Yeo *et al.*, 2011), which revealed two local networks that covered primary cortex (the visual network and the sensorimotor network, which covers both sensorimotor and primary auditory cortex), four distributed association networks (dorsal attention network, ventral attention network, frontoparietal network, and default mode network), and the limbic system (limbic network). Raincloud plots were constructed to compare the distribution of non-zero tumour frequency values between voxels belonging to differing canonical subnetworks.

### Functional connectome

**Figure 2:**
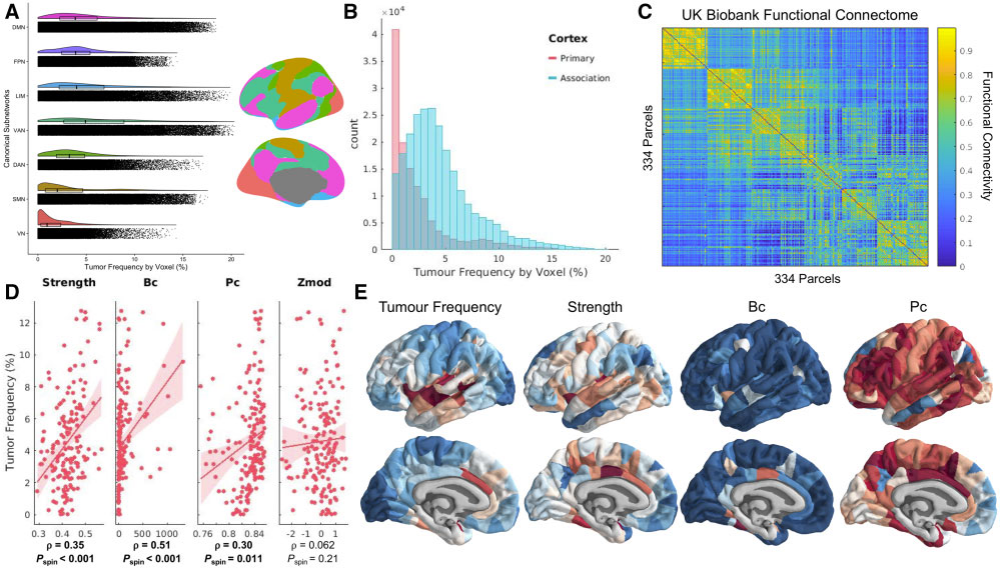
**Gliomas localize to connector hubs of the brain.** (**A**) Raincloud plot comparing glioma frequency distributions across canonical subnetworks. Colour-matched canonical subnetworks are plotted on brain renderings to indicate neuroanatomical positions of these networks. (**B**) Histogram comparing glioma frequency distribution across primary versus association cortex. (**C**) Functional connectome calculated from resting state functional scans of over 4000 UK BioBank participants. Nodes in the network are organized according to their affiliation with different canonical subnetworks. (**D**) Correlations between glioma frequency and hub measures calculated from the functional connectome. (**E**) Visualization of glioma frequency and functional hub measures on the cortical surface. Bc = betweenness centrality; Pc = participation coefficient.

Glioma frequency was compared to regional connectivity (hubness) as quantified by graph theory metrics applied to the functional connectome derived from resting state functional MRI data (Miller *et al.*, 2016; Alfaro-Almagro *et al.*, 2018) from over 4000 neurologically healthy UK BioBank participants (age range 44–78 years; 53% female). The publicly available ‘dense voxel-wise connectome’ of the first UK BioBank cohort (https://www.fmrib.ox.ac.uk/ukbiobank/) corresponds to a 4D image, where each voxel consists of 1200 principal components derived from a group-level principal component analysis (PCA; Smith *et al.*, 2014). A correlation between voxels across components gives a close, memory efficient approximation to the correlation of blood oxygenation level-dependent signal calculated across concatenated time points from all individual participants (Smith *et al.*, 2014). The aforementioned 334 region in-house parcellation, covering both subcortical and cortical regions, was applied to the voxel-wise connectome. PCA loadings for voxels within each parcel (i.e. region) were averaged (analogous to a mean timeseries) and correlated between parcels to produce the weights of a graph (Fig. 2C). Diagonal elements and negative correlations were set to zero. The same parcellation was also applied to the tumour frequency map to quantify tumour frequency within each parcel. Tumour frequency for a parcel represented the average percentage of lesion overlap of voxels within that parcel. The common parcellation allowed for comparison between measures of tumour frequency and functional hubness.

### Graph theory metrics of hubness

Once the weighted healthy connectome had been constructed, we calculated graph theoretical metrics of hubness using the Brain Connectivity Toolbox (Rubinov and Sporns, 2010). In this graph theoretical approach to neuroimaging data, parcels of the brain are conceived as ‘nodes’, whereas correlations in functional activity between parcels are conceived as the weights of connections between the nodes. Hub metrics derived included: nodal strength (sum of all weighted connections for a particular node), betweenness centrality (fraction of all shortest paths in a network that pass through a certain node), clustering coefficient (average weighted connections of triangular subgraphs associated with a node), local efficiency (inverse of the average shortest path length between a node and every other node), eigenvector centrality (the extent to which a given brain region connects to other regions with higher centrality), participation coefficient (the strength of connections outside of a node’s given module relative to connections within that node’s module), and within-module degree *z*-score (nodal strength of a node within its module, compared to within-module nodal strengths of each other node in the module). To reduce the impact of community affiliation on participation coefficient and within-module degree *z*-score, community affiliations were designated based on the maximum spatial overlap of each node with one of the seven canonical subnetworks (Yeo *et al.*, 2011).

Hub measures were calculated for each of the 334 nodes of the functional connectome. Measures from homotopic nodes were then averaged together, resulting in 167 observations for each subcortical and cortical parcel per hub metric. Many of the hub measures were observed to have a high correlation with nodal strength. Therefore, we screened out hub measures that had a Spearman’s correlation of ρ > 0.95 with nodal strength. This led to the removal of clustering coefficient, local efficiency, and eigenvector centrality. While this threshold is arbitrary, the same result was reached with thresholds ranging from 0.65 to 0.99 (Supplementary Fig. 3). Spearman’s correlations were calculated between the remaining hub metrics and tumour frequency and were assessed for significance by comparison to spatially contiguous null models, via the spin test (Supplementary material).

### Cellular correlates of tumour frequency

To determine whether tumours were more common in regions enriched for neural stem cells, we assessed tumour frequency within the two parcels of our 334 region parcellation which most closely aligned with the subventricular zone and the dentate gyrus: the caudate and the hippocampus. The average tumour frequency between these two parcels was compared to average tumour frequency between 10 000 random pairs of parcels.

To determine if tumours were more common in regions enriched for OPCs, we compared tumour frequency to an expression map of OPC cell class. This expression map was estimated by assessing transcriptional enrichment of OPC genetic markers using a procedure analogous to that previously reported (Seidlitz *et al.*, 2020) and described in more detail in the Supplementary material. In summary, transcriptional enrichment of an OPC gene set was assessed at each cortical brain parcel using the publicly available Allen Human Brain Atlas (AHBA; Hawrylycz *et al.*, 2012). The OPC gene set was derived from a separate single cell RNA sequencing study that distinguished post-mortem human cortical cells by canonical cell types (Lake *et al.*, 2018). We confirmed the spatial specificity of this gene set by comparing its co-expression pattern with 1000 identically sized sets of randomly chosen genes. Genes that did not share a positive co-expression pattern with the overall group were filtered out. Median regional enrichment of the resulting gene set was then calculated for each cortical parcel, correlated with tumour frequency, and tested for significance using the spin test. 

### Aligning tumour frequency map with the Allen Human Brain Atlas

We compared tumour frequency with post-mortem gene expression from the AHBA (http://human.brain-map.org/; Hawrylycz *et al.*, 2012). The AHBA catalogues post-mortem gene expression from six individuals (aged 24 to 57 years old; five males and one female; deceased from non-neurologically related causes) at a variety of brain locations. Preprocessing of the AHBA data followed a similar pipeline to previous work from our group (Romero-Garcia *et al.*, 2018, 2019) and is described in more detail in the Supplementary material. Transcription levels for 20 647 genes across 2748 sample locations were related to tumour frequency at each sample location using partial least squares (PLS) regression. Tumour frequency values were aligned with sample locations by warping the non-smoothed, non-mirrored tumour frequency map into the standard stereotactic space of the Montreal Neurological Institute (MNI), a standard brain template for which the locations of the AHBA microarray samples are known. Once in MNI space, a 2 mm FWHM smoothing kernel was applied to the map and the map was mirrored to the left hemisphere. Sample locations from the AHBA that were located in the right hemisphere were also mirrored to their homotopic voxel in the left hemisphere. This alignment resulted in a 2748 (samples) × 20 647 (genes) expression matrix and in a vector of 2748 elements representing tumour frequency values matched to each sample’s MNI coordinates (Fig. 4A and B). Tumour frequency values were square rooted to reduce the skewness of the tumour frequency distribution (Fig. 1B). Gene expression values were *Z*-scored for each gene. To test the robustness of the findings, the analyses below were repeated using tumour frequency maps derived from Groups 1 and 2.

### Transcriptomic correlates of tumour frequency

PLS regression was used to relate spatial transcription patterns of 20 647 genes with the spatial distribution of glioma. PLS regression involves projecting a predictor (*x*) and a response (*y*) matrix into a space where linear combinations of *x* explain the maximum amount of variance in *y*. We chose to focus on the first two components from PLS (PLS1 and PLS2) as the subsequent components explained a proportion of variance indistinguishable from one another (Supplementary Fig. 4A). Because PLS is a supervised learning technique, the significance of the model cannot be accurately estimated from the relationships between PLS components and the dependent variable. Therefore, following a previously described approach (Whitaker *et al.*, 2016), statistical significance of the PLS model was tested via permutation testing, by comparing the per cent variance explained in the original model to a distribution of 1000 models where the sample labels mapping *x* to *y* were randomly shuffled. Significance of each PLS coefficient was tested via bootstrapping with 1000 iterations, resulting in two *Z*-statistics for each gene, one for the first PLS component and another for the second PLS component. Genes were ranked by their *Z*-statistics and entered into gene ontology analyses in GOrilla (http://cbl-gorilla.cs.technion.ac.il/), resulting in a hierarchy of biological terms associated with each PLS component, visualized using Revigo (Supek *et al.*, 2011). To ensure a data-driven approach, genes with *Z*-statistic values that did not meet the Bonferroni-corrected significance threshold were not excluded from the gene lists.

### Relating partial least squares components to glioma proto-oncogenes

We sought to determine whether either of our PLS components were enriched for genes that are dysregulated in glioma. We collected a list of 17 proto-oncogenes from a recent review (Molinaro *et al.*, 2019; Supplementary material) that are known to be either mutated, amplified, or lost in specific subtypes of glioma. 

Similar to the OPC gene list preprocessing, we first confirmed that these genes co-expressed significantly (compared to 10 000 identically sized sets of genes). Next, we filtered out genes with differing co-expression patterns from the group (denoted by negative correlations, on average, with other genes in the set), leading to the exclusion of three genes (*IDH2*, *MYCN*, and *CIC*). The median rank of the final list of 14 genes was determined among the first and second PLS components and assessed for significance by comparison to median ranks expected by chance.

### Visualization of partial least squares components

We were interested in the locations of the samples that drove each PLS component. First, PLS1 and PLS2 loadings were plotted and coloured based on the affiliation of the sample with cortex or subcortex. To determine how PLS1 and PLS2 loadings mapped onto cortex, we assigned samples to parcels via a nearest neighbour mapping. Then, the PLS loading of a parcel was represented as the median PLS loading across samples assigned to that parcel. Two parcels were assigned zero samples from nearest neighbour mapping, and these parcels were assigned the mean loading of the group. More samples were mapped to each subcortical parcel [*n *=* *8; mean = 78.9; standard deviation (SD) = 52.1] compared to cortical parcels (*n *=* *159; mean = 13.3; SD = 12.5).

### Multivariate model combining connectomic, cellular and genetic contributions to tumour frequency

To determine how different measures of biological contributions to glioma risk interrelated, we developed a multiple linear regression model combining each of the factors we found to be associated with tumour frequency. The model included nodal strength, OPC distribution, PLS1 loadings, and PLS2 loadings. Each of these measures was represented as a 167-dimensional vector, with a value ascribed to each parcel within our parcellation scheme. The dependent variable for the model was the square root of average tumour frequency within each parcel, addressing the skewness of the original tumour frequency values (Fig. 1B). The dependent variable and each of the predictors were *Z*-scored, and zeros were assigned to parcels for which no value could be appropriately calculated (e.g. subcortical parcels for OPC distribution, and parcels mapped to zero samples for PLS1 and PLS2 loadings).

First, we constructed a model to determine whether there were any two-way interaction effects between the different scales of biological factors. Nodal strength represented ‘connectomic factors’, OPC distribution represented ‘cellular factors’, and PLS1 and PLS2 loadings represented ‘genetic factors’. This model had the following form:

(1) $${\mathrm{tumou}r_{\;}\mathrm{frequency}}_{i}=b0+b1\;\times \;\mathrm{str}e\mathrm{ngth}+b2\;\times \;\mathrm{OPC}+b3\;\times \;\mathrm{PLS}1+b4\;\times \;\mathrm{PLS}2+b5\;\times \;\mathrm{strength}\left|\mathrm{OPC}+b6\;\times \;\mathrm{strength}\right|PLS1+b7\;\times \;\mathrm{strength}\left|\mathrm{PLS}2+b8\;\times \;\mathrm{OPC}\right|PLS1+b9\;\times \;\mathrm{OPC}|\mathrm{PLS}2+\varepsilon$$

where *tumour frequency_i_* is the square root of the tumour frequency at parcel *i*. This model revealed no significant interaction effects between different biological factors. Therefore, we constructed a second model with no interaction terms, of the form:

(2) $${\mathrm{tumou}r_{\;}\mathrm{frequency}}_{i}=b0+b1\;\times \;\mathrm{strength}+b2\;\times \;\mathrm{OPC}+b3\;\times \;\mathrm{PLS}1+b4\;\times \;\mathrm{PLS}2+\varepsilon$$

After determining the percentage of explained variance in tumour frequency from these predictors, we explored the individual contribution of each variable by calculating the square of the partial correlation between that variable and tumour frequency. Significance of the explained variance was assessed by comparison to the distribution of explained variances between the variable and 10 000 permuted, spatially contiguous, null models of tumour frequency.

### Statistical analyses

Statistical analyses and preprocessing of all neuroimaging and genetic data were performed in MATLAB 2017b. All brain map comparisons were conducted using inter-parcel Spearman rank-correlations, while significance was determined using a non-parametric permutation test. Permutation tests were also used to test glioma localization to NSC niches, spatial specificity of gene list expression, significance of PLS model, and enrichment of PLS-ranked gene lists for glioma proto-oncogenes. Normality assumptions for the PLS and multiple linear regression models were assessed via visual inspection of the distributions for each variable, which were transformed when necessary. Spatial autocorrelation of each brain map was investigated using Moran’s *I* (Supplementary material). Data were visualized using BrainsForPublication (https://github.com/WhitakerLab/BrainsForPublication), RainCloudPlots (https://github.com/RainCloudPlots/RainCloudPlots; Allen *et al.*, 2019), and the Gramm toolbox (https://github.com/piermorel/gramm; Morel, 2018).

### Data availability

The anonymized neuroimaging and genetic data described in this study are publicly accessible.

## Results

### Anatomical mapping of glioma distribution

We constructed a map of glioma distribution from aligned masks of tumour volume across 335 high and low grade glioma patients. This tumour frequency map displayed a hemispherically symmetric, but heterogeneous spatial distribution (Fig. 1A and Supplementary Fig. 1). Consistent with prior reports, gliomas were rare in the occipital lobe, but relatively common in the insular cortex (Fig. 1B and Supplementary Table 1). Visual and quantitative inspection of the glioma distribution revealed significant spatial autocorrelation (Fig. 1A, Supplementary Table 2 and Supplementary Fig. 2), prompting the need for spin test methodology to infer brain map correspondence in later analyses. Tumour frequency distributions were replicable across independent, randomly assigned subsets of half of the images (Groups 1 and 2) with an interregional correlation of *r *=* *0.83 (95% CI: *r *=* *0.70–0.93). Replicability of subsequent analyses was tested with Groups 1 and 2 tumour frequency maps (Supplementary material).

Tumour frequency was compared across canonical, large-scale functional networks and primary versus association cortex. Association regions responsible for consolidating information across multiple sensory modalities showed higher tumour prevalence (average voxel: 4.57%) than visual and somatosensory primary cortices, which had the lowest tumour frequency (2.45%), particularly in the visual cortex (1.56%; Fig. 2A and B).

### Gliomas localize to hubs of high connectivity and centrality

Graph theory measures were calculated from the mean functional connectome derived from resting state functional MRI scans of over 4000 UK BioBank participants, and then compared with glioma frequency. The connectome was first organized into seven communities of interconnected nodes based on their overlap with previously defined large-scale functional networks (Yeo *et al.*, 2011) resulting in a neat modular organization, with strong connections within modules and sparse connections between modules (Fig. 2C). Graph theory measures of hubness were then calculated, measuring properties such as connectivity with neighbouring nodes, involvement in shortest paths across the network, connectivity to nodes in different modules, and within-module connectivity (see ‘Materials and methods’ section for how these measures were defined and selected). The interregional correlation between measures of hubness and glioma frequency was tested for significance by comparison with spatially contiguous null models (Fig. 2D).

Glioma frequency strongly correlated with the simplest measure of hubness, nodal strength (ρ  =  0.34; *P*_spin_ = 0.00055), which aggregates the weights of a node’s immediate connections. Glioma frequency was also significantly correlated with betweenness centrality (ρ  =  0.51; *P*_spin_ = 0.0002) and with a measure of connectivity to diverse communities, the participation coefficient (ρ  =  0.30; *P*_spin_ = 0.011). Connectivity within a community, measured by Z-score modularity, did not relate with glioma frequency (ρ  =  0.062; *P*_spin_ = 0.21; Fig. 2D). This profile of connectivity measures was most consistent with that of connector hubs that link together multiple subnetworks.

### Glioma frequency is elevated in areas with populations of stem-like brain cells

**Figure 3:**
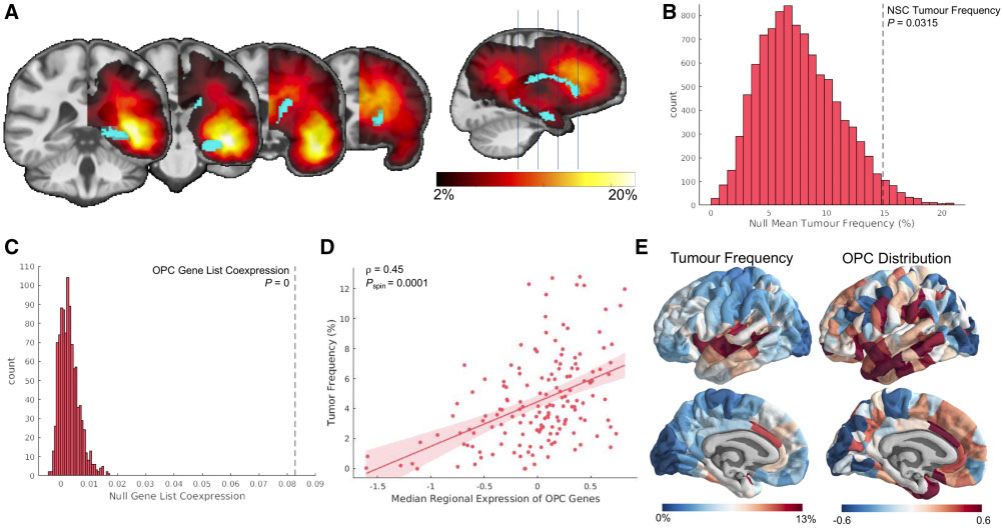
**Gliomas localize to brain regions enriched with stem-like cells.** (**A**) Visualization of the parcel masks representing the hippocampus and caudate superimposed on the mirrored tumour frequency map. (**B**) Average tumour frequency across the hippocampus and caudate (represented as the dotted black line) compared to a distribution of average tumour frequency across 10 000 sets of two randomly chosen parcels. (**C**) Co-expression among genes within the OPC gene list compared to co-expression among 10 000 identically-sized sets of genes. (**D**) Correlation between OPC distribution across cortex and glioma frequency (ρ  =  0.45; *P*_spin_ = 0.0001). (**E**) Visualization of glioma frequency and OPC distribution on the cortical surface.

We tested the hypothesis that brain regions enriched with neural stem cells were more likely to coincide with loci of high frequency of gliomas. Mean tumour frequency was calculated from the hippocampus and the caudate (Fig. 3A), regions that best approximate the locations of the only known sources of neural stem cells in the adult human brain. Tumour frequency across bilateral hippocampus and caudate were averaged, and compared against a null distribution of 10 000 different pairs of randomly selected parcels within our 334-region parcellation scheme. Glioma frequency was observed to be significantly higher in these two regions compared to the null distribution (*P *=* *0.0315; Fig. 3B).

Next, we tested the spatial correspondence of glioma distribution with the patterning of OPCs, which are also hypothesized to be cells of origin for glioma. OPC distribution was estimated from the expression of genetic markers of OPC identity using post-mortem the microarray data of the AHBA (www.brain-map.org). The list of genetic markers for OPCs co-expressed significantly (Fig. 3C), confirming that median expression across this gene list represents a spatially specific phenotype. This estimate of OPC patterning correlated significantly with glioma frequency (Fig. 3D; ρ  =  0.45; *P*_spin_ = 0.0001).

### Transcriptomic correlates of glioma frequency

**Figure 4:**
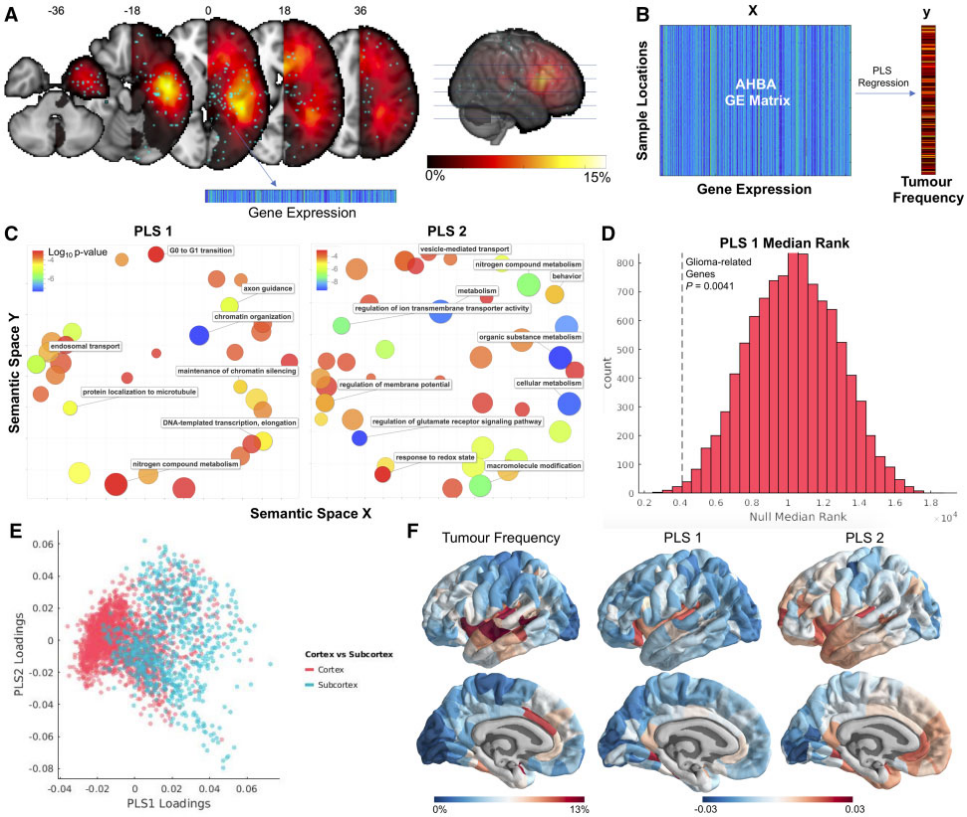
**Transcriptomic correlates of glioma frequency.** (**A**) Alignment of AHBA sample locations to the tumour frequency map. (**B**) Illustrative flow chart of the statistical analysis relating normative spatial gene expression patterns to glioma frequency. (**C**) Gene ontology terms associated with two PLS components (PLS1 and PLS2) that related gene expression with glioma frequency. (**D**) Rank of 14 glioma proto-oncogenes compared with null distribution of median ranks. (**E**) AHBA samples plotted by PLS1 loadings, PLS2 loadings, and cortex versus subcortex. (**F**) Visualization of glioma frequency, PLS1 loadings, and PLS2 loadings on the cortical surface. PLS loadings from samples were assigned to parcels via a nearest neighbour mapping.

We used PLS regression to relate the spatial transcription patterns of 20 647 genes with tumour frequency at 2748 cortical and subcortical locations where gene expression was assessed in post-mortem adult human brain tissue (Fig. 4A and B). The first two components of the PLS (PLS1 and PLS2) explained 19% and 18% of the tumour frequency variance, respectively (Supplementary Fig. 4A). The total variance explained by the model was significantly greater than equivalent PLS models trained on random permutations of the data (permutation test; *P *<* *0.001; Supplementary Fig. 4B).

Bootstrapping was performed on PLS weights resulting in *Z*-statistics for each gene corresponding to the PLS1 and PLS2 ranking (Supplementary Fig. 4C). The ranked gene lists were entered into a gene ontology (GOrilla; http://cbl-gorilla.cs.technion.ac.il/). Genes corresponding to PLS1 were related to biological processes such as chromatin organization, endosomal transport, and G0 to G1 transition. Genes corresponding to PLS2 were related to a broad set of metabolic processes along with many components of synaptic transmission (Fig. 4C). PLS1 was also found to be significantly enriched for genetic drivers of gliomagenesis (*P *=* *0.0041; Fig. 4D). PLS2 was not significantly enriched for this set of genes (*P *=* *0.64).

PLS1 was more highly loaded onto the subcortex relative to the cortex (Fig. 4E). PLS loadings for each AHBA sample were mapped to their nearest brain region for visualization on the cortical surface (Fig. 4F).

### Connectomic, cellular and genetic contributions to glioma frequency are independent

**Figure 5:**
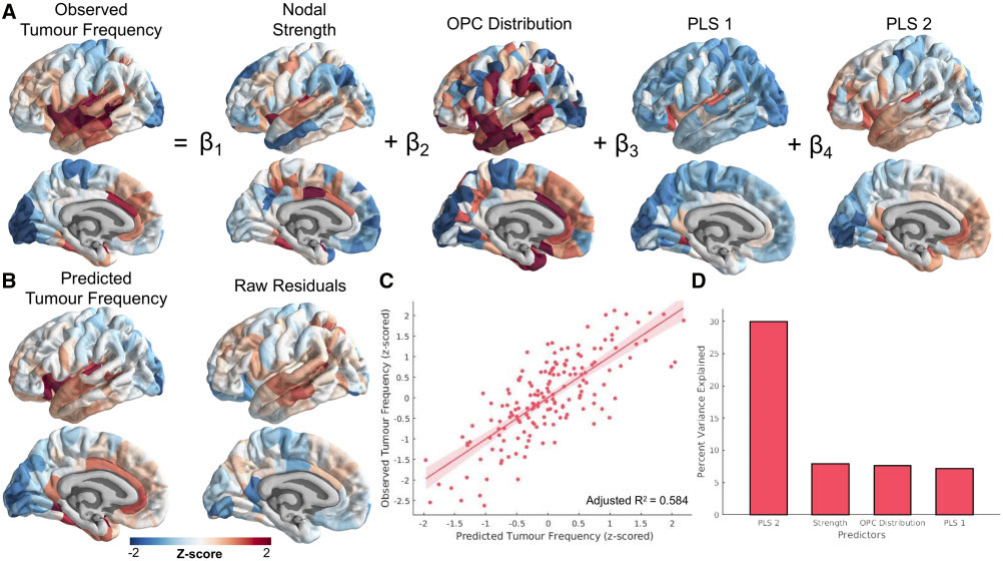
**Multiple linear regression model relating connectomic, cellular and transcriptomic factors with glioma distribution.** (**A**) Schematic of the multiple linear regression model. Intercept and error terms are not displayed. (**B**) Fitted values and residuals of glioma distribution model. (**C**) Scatter plot of predicted versus observed tumour frequency values. (**D**) Per cent of variance explained by each individual predictor of tumour frequency. These values were calculated using the partial correlation coefficient between each measure and tumour frequency.

Finally, we sought to reveal the interrelations between the connectomic, cellular, and genetic contributions to glioma distribution uncovered in the study. A multiple linear regression model was constructed, with factors of nodal strength, OPC distribution, PLS1 loadings, and PLS2 loadings (Fig. 5A). NSC distribution was not included in the model because this measure could not be quantified at each brain parcel. First, we tested a model to determine if there were interaction effects between connectomic (nodal strength), cellular (OPC distribution), and genetic (PLS1 and PLS2 loadings) factors. None of the interaction effects were significant. The model without interaction effects explained ∼58% of the variance in glioma frequency [*F*(4,162) = 59.3; *P *=* *9.37 × 10^−31^; adjusted R^2^ = 0.584; Fig. 5B and C). All individual factors significantly predicted tumour frequency variance (Table 1 and Fig. 5D). Because of the unequal mapping of AHBA samples to cortical versus subcortical regions, the PLS2 component, which is more highly represented across cortex, explained more of the variance in tumour frequency than PLS1 once projected onto the anatomy. It is also worth noting that the amount of variance explained by the PLS factors was inflated by construction, due to the large number of input variables and the design of the technique which results in maximizing covariance (Mufford *et al.*, 2017).

**Table 1:** Results of multiple linear regression model predicting tumor frequency

Predictor	β-value	Standard error	*T*-statistic	% Explained variance	Spin test, corrected *P*-value
Intercept	3.2 × 10^−16^	0.0499	6.37 × 10^−15^	NA	NA
Nodal strength	0.202	0.0543	3.72	7.86	0.0010
OPC distribution	0.206	0.0566	3.64	7.58	0.0040
PLS1 loadings	0.210	0.0594	3.54	7.17	0.0052
PLS2 loadings	0.480	0.0577	8.32	29.9	0

## Discussion

In this study, we examined the network, cellular, and transcriptomic correlates of brain regions commonly frequented by glioma to test specific hypotheses regarding gliomagenesis. We found that gliomas were most common in association cortex and connector hub regions. Elevated glioma frequency was observed in brain regions expected to be populated by neural stem cells and OPCs. Finally, we determined that glioma distribution correlated with the spatial transcription patterns of genes related to metabolic activity, synaptic signalling, and gliomagenesis. These findings support the predictions of network neuroscience and cancer theory, and establish links between concepts from these two frameworks to characterize the spatial distribution of adult gliomas.

### Localization of neurological disease to brain hubs

An extensive body of work has demonstrated the utility of network models in predicting the spread of disease (Zhou *et al.*, 2012; Brown *et al.*, 2019; Henderson *et al.*, 2019) as well as the vulnerability of particular brain regions to disease (Seeley *et al.*, 2009; Crossley *et al.*, 2014; Warren *et al.*, 2014). In this work, we used network models to demonstrate for the first time that functional hub regions of the brain are vulnerable to the concentration of gliomas. In particular, gliomas appear to localize to brain regions expected to play the role of connector hubs, nodes that link diverse cognitive subsystems with one another, as opposed to provincial hubs, which integrate communication within their own subsystems (van den Heuvel and Sporns, 2013). Consistent with this idea, gliomas were more common in association cortical regions important for consolidating information across sensory modalities (Mesulam, 2015). This suggests that the brain regions that facilitate long distance connections across the cortex are especially vulnerable to oncogenesis, consistent with our hypothesis that the high metabolic cost of such connections influences glioma risk (Bullmore and Sporns, 2012).

Alternatively, the results can be interpreted as reflecting a higher likelihood for tumour infiltration of hub regions. Gliomas are known to migrate throughout the brain via blood vessels and white matter tracts, which contributes to the poor prognosis of glioblastoma multiforme. Here we consider only the location of the tumour during the preoperative scan, which could represent either the tumour origin, or to where it spread during the progression of the disease. Although networks were constructed from functional MRI and not white matter tracts along which tumours are known to infiltrate (Pedersen *et al.*, 1995), recent work on activity-dependent glioma migration suggests that tumours could preferentially invade functional hubs (Venkatesh *et al.*, 2019). Because of their high centrality, hubs are, by definition, likely to be encountered during random walks within a network.

### Cellular origins of glioma

Early work on gliomagenesis hypothesized that mature glial cells were the cells of origin for adult glioma. However, it was soon recognized that the cell of origin most likely maintains pluripotency after development, since such cells require fewer mutations to become cancerous (Sanai *et al.*, 2005). Following recent evidence of neural stem cells in the subventricular zone and dentate gyrus of the hippocampus of adults, there is an emerging consensus that stem-like cells could be the cells of origin for glioma (Sanai *et al.*, 2005; Jiang and Uhrbom, 2012). Recent work has provided evidence that some isocitrate dehydrogenase (IDH) wild-type glioblastomas originate from stem cells in the subventricular zone (Lee *et al.*, 2018). Lee and colleagues demonstrated that for a majority of their glioblastoma patients, the unaffected subventricular zone carried low-level driver mutations, which were present to a greater extent in the tumour. Our findings complement this research by establishing that gliomas in general are more highly concentrated in regions enriched with neural stem cells.

OPCs have also been hypothesized to represent cells of origin for glioma. Evidence for this idea comes from studies demonstrating that some gliomas express OPC genetic markers (Shoshan *et al.*, 1999; Verhaak *et al.*, 2010), and that OPCs can be experimentally manipulated into becoming cancer stem cells (Kondo and Raff, 2000; Liu *et al.*, 2011). OPCs comprise the majority of dividing cells in the adult brain and are distributed broadly throughout the subventricular zone, white matter, and grey matter (Jiang and Uhrbom, 2012; Hughes *et al.*, 2013). We estimated this distribution by quantifying normative expression levels of OPC genetic markers across the human brain, and found that it significantly correlated with glioma frequency. While this result aligns nicely with prior work, estimates of OPC distribution relied on combining data from two independent transcriptomic studies of post-mortem human brains (Hawrylycz *et al.*, 2012; Lake *et al.*, 2018). While this approach has been validated for determining the brain-wide distribution of other canonical cell types (Seidlitz *et al.*, 2020), our result should be confirmed once more reliable estimates of OPC patterning become available.

### Genetic determinants of glioma vulnerability

Normal cells can become malignant through a series of somatic mutations which disable tumour suppressors and activate drivers of cell proliferation (Mukherjee, 2010). To determine the genetic alterations involved in oncogenesis, much research has focused on identifying molecular genetic differences between tumour cells and matched healthy tissue (McLendon *et al.*, 2008; Tang *et al.*, 2018). Here, we took an alternative approach and investigated transcriptomic differences between healthy regions where tumours tend to occur versus healthy regions where tumours are uncommon. This approach recapitulated prior research into glioma genetics, in that genes which drive gliomagenesis appeared to be upregulated among the healthy transcriptomic correlates of glioma distribution. Gene ontology revealed that the genes driving PLS1 (the component responsible for most of the covariance between transcription and glioma distribution) were most strongly associated with chromatin organization, a process perturbed by IDH mutations and critically involved in the pathogenesis of glioma (Suzuki *et al.*, 2015; Reifenberger *et al.*, 2017). In addition, our approach also revealed novel findings, such as the carcinogenic vulnerability of healthy brain regions enriched with genes coordinating synaptic signalling and metabolic activity. These findings complement our connectomic results, providing more evidence for the idea that metabolically demanding brain regions crucial for brain-wide communication are susceptible to oncogenesis.

The goal of this study was to examine brain regions generally implicated in adult glioma. However, glioma is a heterogeneous phenomenon, comprising tumours of differing genetic aetiologies and morphologies. It is known that different types of glioma tend to localize to different brain regions (Zlatescu *et al.*, 2001; Mueller *et al.*, 2002; Duffau and Capelle, 2004; Tejada Neyra *et al.*, 2018). Therefore, the exact composition of patients (e.g. proportion of high grade to low grade glioma patients) within our sample could influence the results. To address this concern, we replicated our results with subgroups of varying proportions of high grade to low grade glioma patients and demonstrated that our results are robust to changes to the composition of the sample. However, we did not have access to the molecular genetic characterization of the tumours in our sample, limiting our ability to determine the effect of tumour genotype on the results. Examining glioma subtypes separately could illuminate the network, cellular, and transcriptomic correlates which distinguish localization patterns of different types of glioma. Such work could be useful for developing scientifically informed priors for tumour diagnosis before biopsy, so this question is of both scientific and clinical interest.

## Conclusion

Gaining a better understanding of the mechanisms driving glioma localization patterns could provide a more detailed account of the aetiology of the disease and consequently inform treatment targets. We demonstrated that glioma distribution can in part be explained by functional hubness, distribution of stem-like cells, and transcription patterns of genetic determinants of glioma. These results add to previous literature reporting the vulnerability of hub regions to neurological disease (Bullmore and Sporns, 2012; Crossley *et al.*, 2014) and provide support for cancer stem cell theories of glioma (Sanai *et al.*, 2005; Ma *et al.*, 2009; Visvader, 2011). Our findings highlight the importance of bridging diverse scales of biological organization in the study of neurological dysfunction.

## Supplementary Material

awaa277_Supplementary_Data

## Abbreviations

AHBA =: Allen Human Brain Atlas
OPC =: oligodendrocyte precursor cell
PLS =: partial least squares
