# Supplementary material for: Genetic, cellular, and connectomic characterization of the brain regions commonly plagued by glioma
Source: Brain. 2020 Dec 4;143(11):3294–307. doi: 10.1093/brain/awaa277 (PMC7891236; doi:10.1093/brain/awaa277)
Supplement: awaa277_Supplementary_Data [file awaa277_supplementary_data.pdf]

Genetic, Cellular, and Connectomic Characterization of Adult Human Brain Regions Commonly Plagued by Glioma

Supplemental Information

Ayan S. Mandal[1*], Rafael Romero-Garcia[1], Michael G. Hart[1,2], John Suckling[1]

[1]Brain Mapping Unit, Department of Psychiatry, University of Cambridge

[2]Academic Division of Neurosurgery, Department of Clinical Neurosciences, University of Cambridge

* Correspondence to: Ayan Mandal, University of Cambridge, Department of Psychiatry, Herchel Smith Building, Robinson Way, Cambridge, UK CB2 0SZ, Email: asm82@cam.ac.uk

Supplementary Methods

Spin Test Methodology

The "spin test" involves comparing the observed inter-parcel correlation between maps of two measures with a distribution of the correlations calculated after one of these maps has been spatially permuted in a way that preserves contiguity between brain regions. Spatial permutation was accomplished by projecting the centroid coordinates for each parcel onto an inflation of the pial surface as a sphere (Fischl, 2012), applying a random rotation to that sphere, and then projecting the new coordinates back onto the pial surface and assigning them to the nearest centroid coordinates of the original parcellation. The result is a shuffled parcellation where most parcels remain contiguous.

Past studies using the spin test have focused on comparisons between cortical brain maps. However, subcortical regions were also of interest in this study. Subcortical regions cannot be projected onto the inflated spherical pial surface, so an alternative approach was needed. We incorporated the subcortex into our null models by shuffling the eight subcortical regions with respect to one another, whereas the cortical regions were shuffled using the spin test.

After each spin permutation, two correlations were calculated; one between measures estimated from parcels in their original configuration and the other in its permuted configuration, and vice versa. These two correlations were averaged to form one of the 10000 values forming a null distribution to which the observed correlation was compared to determine statistical significance, as the proportion of null correlations greater than the observed correlation (i.e. P_{spin}).

AHBA Preprocessing

Custom microarrays were used to measure the expression of all genes in the genome in 3702 brain sample locations across cortex, subcortex, and cerebellum (Hawrylycz *et al.*, 2012). Pre-processing of these data followed a similar pipeline to previous work from our group (Romero-Garcia *et al.*, 2018, 2019). Microarray probes were mapped to genes using the genome assembly hg19 (UCSC GenomeBrowser; http://sourceforge.net/projects/reannotator/; Arloth *et al.*, 2015). In line with criteria from Richiardi and colleagues (Richiardi *et al.*, 2015), probes were matched to a gene only if there were less than three mismatches between the probe and reference

sequence. When a gene matched with multiple probes, the probe with the highest average expression across samples was selected to represent the expression patterns of that gene. A recent study demonstrated the effectiveness of this preprocessing step in increasing the correspondence between microarray and RNA-seq expression (Arnatkevičiūtė *et al.*, 2019). In total, the expression patterns of 20647 genes across each sample location were evaluated. Samples which were collected from the brain stem and cerebellum were excluded from the analysis, leading to a final number of 2748 samples.

Estimation of OPC Distribution

The estimation of OPC distribution followed three steps: (i) selection of a set of genes associated with OPC identity, (ii) filtering of this gene set to allow for integration with the AHBA, and (iii) assessment of median regional enrichment of OPC associated genes. First, an OPC gene set was derived from a single cell RNA sequencing study performed on adult postmortem cortical tissue (Lake *et al.*, 2018) that determined genes with transcription patterns distinguishing cells by canonical cell types, including excitatory and inhibitory neurons, astrocytes, oligodendrocytes, and OPCs. The set of 132 genes that distinguished OPCs from other canonical cell classes across the cortex was downloaded from previously published material (Lake *et al.*, 2018).

Next, we determined regional OPC enrichment in the adult brain using the publicly available Allen Human Brain Atlas (AHBA; Hawrylycz *et al.*, 2012). Transcription patterns of 20,647 genes were aligned to the 159 left hemisphere cortical regions in our parcellation, using prior methods (Romero-Garcia *et al.*, 2018, 2019) with code available for download (https://github.com/RafaelRomeroGarcia/geneExpression_Repository). The resulting 159 x 20,647 regional gene expression matrix was z-scored by parcel. Because the OPC gene set was derived from sequencing performed on cortical brain tissue, we decided to exclude subcortical regions from this part of the analysis. 13 genes in the OPC gene set were not matched to any AHBA probe and were consequently excluded from the analysis. We evaluated the spatial specificity of the remaining 119 OPC genes by comparing their co-expression pattern with 1000 identically-sized sets of randomly chosen genes. OPC genes that did not share a positive co-expression pattern with the overall group of genes were filtered out. Concretely, the 24 genes which had, on average, negative correlations with other genes in the set were removed from the

OPC gene set. We estimated OPC distribution by calculating the median regional enrichment of the filtered OPC gene set across cortical parcels. OPC distribution across 159 cortical parcels was then correlated with tumor frequency and tested for significance using the spin test.

Supplementary Results

Influence of spatial vicinity on brain maps

To determine the degree to which spatial proximity explained the variance in brain maps, we computed the Moran's I of each key variable considered in the study. Moran's I is a normalized ratio of the covariance among spatially vicinal regions to the variance of the total brain map (Moran, 1950). Adjacent brain regions within the 167-region parcellation were categorized as vicinal, using a spatial weight matrix W, where element $W_{ij} = 1$ if parcels i and j are neighbors and 0 otherwise. Moran's I for each variable is displayed in Supplementary Table 2. Each of these variables exhibited significant (non-random) spatial autocorrelation (Permutation test; $p < 0.001$; Supplementary Figure 2).

Replication of main findings

To determine the robustness of the results, major findings were internally replicated by using sub-cohorts of patients with varying proportions of high- and low-grade glioma. Similar to the connectomic results from the full cohort, glioma frequency derived from Group 1 was associated with nodal strength (rho = 0.37; $P_{\text{spin}} = 0.00025$), betweenness centrality (rho = 0.48; $P_{\text{spin}} = 0.0002$), and participation coefficient (rho = 0.34; $P_{\text{spin}} = 0.0042$), but not Z-score modularity (rho = 0.033; $P_{\text{spin}} = 0.33$), while glioma frequency derived from Group 2 was associated with nodal strength (rho = 0.29; $P_{\text{spin}} = 0.00033$), betweenness centrality (rho = 0.51; $P_{\text{spin}} = 0.0002$), and participation coefficient (rho = 0.25; $P_{\text{spin}} = 0.027$), but not Z-score modularity (rho = 0.072; $P_{\text{spin}} = 0.19$). The association between OPC distribution and glioma frequency was also internally replicated: Group 1: rho = 0.41; $P_{\text{spin}} = 0.0005$; Group 2: rho = 0.46; $P_{\text{spin}} = 0.0001$. Finally, PLS1 and PLS2 genes lists from the full cohort correlated with gene lists from Group 1 at rho = 0.991 and rho = 0.989 respectively, and with Group 2 at rho = 0.992 and rho = 0.990. PLS1 and PLS2 gene lists from Group 1 and Group 2 correlated with one another at rho = 0.967 and rho = 0.958

Parcel Names	% Tumor Frequency	Parcel Names (cont.)	% Tumor Frequency (cont.)
Thalamus-Proper	3.7	pericalcarine_part3	0.0
Caudate	8.5	postcentral_part1	0.9
Putamen	8.8	postcentral_part2	6.9
Pallidum	6.5	postcentral_part3	1.6
Hippocampus	9.0	postcentral_part4	2.6
Amygdala	8.5	postcentral_part5	2.8
Accumbens-area	4.9	postcentral_part6	1.8
VentralDC	2.7	postcentral_part7	1.7
bankssts_part1	8.0	postcentral_part8	1.9
bankssts_part2	12.5	posteriorcingulate_part1	3.5
caudalanteriorcingulate_part1	10.8	posteriorcingulate_part2	4.0
caudalmiddlefrontal_part1	6.0	precentral_part1	1.3
caudalmiddlefrontal_part2	6.1	precentral_part2	6.3
caudalmiddlefrontal_part3	3.3	precentral_part3	1.1
caudalmiddlefrontal_part4	4.3	precentral_part4	4.3
cuneus_part1	0.1	precentral_part5	1.4
cuneus_part2	1.5	precentral_part6	3.4
cuneus_part3	0.3	precentral_part7	2.4
entorhinal_part1	6.7	precentral_part8	2.5
fusiform_part1	4.2	precentral_part9	3.5
fusiform_part2	6.3	precuneus_part1	2.2
fusiform_part3	1.0	precuneus_part2	3.9
fusiform_part4	9.4	precuneus_part3	4.4
fusiform_part5	3.4	precuneus_part4	2.1
fusiform_part6	6.8	precuneus_part5	3.3
inferiorparietal_part1	2.6	precuneus_part6	2.3
inferiorparietal_part2	8.1	precuneus_part7	3.7

inferiorparietal_part3	3.8	rostralanteriorcingulate_part1	7.5
inferiorparietal_part4	6.4	rostralmiddlefrontal_part1	5.0
inferiorparietal_part5	4.4	rostralmiddlefrontal_part2	5.3
inferiorparietal_part6	4.2	rostralmiddlefrontal_part3	2.3
inferiorparietal_part7	4.4	rostralmiddlefrontal_part4	5.9
inferiorparietal_part8	3.3	rostralmiddlefrontal_part5	3.6
inferiorparietal_part9	3.6	rostralmiddlefrontal_part6	6.0
inferiortemporal_part1	5.2	rostralmiddlefrontal_part7	4.6
inferiortemporal_part2	3.5	rostralmiddlefrontal_part8	7.3
inferiortemporal_part3	8.4	rostralmiddlefrontal_part9	5.1
inferiortemporal_part4	7.7	rostralmiddlefrontal_part10	5.5
inferiortemporal_part5	4.3	rostralmiddlefrontal_part11	5.4
inferiortemporal_part6	5.9	superiorfrontal_part1	1.5
isthmuscingulate_part1	2.7	superiorfrontal_part2	2.9
isthmuscingulate_part2	4.3	superiorfrontal_part3	3.1
lateraloccipital_part1	1.4	superiorfrontal_part4	4.0
lateraloccipital_part2	4.7	superiorfrontal_part5	2.4
lateraloccipital_part3	3.2	superiorfrontal_part6	6.6
lateraloccipital_part4	0.3	superiorfrontal_part7	3.0
lateraloccipital_part5	0.8	superiorfrontal_part8	7.9
lateraloccipital_part6	2.7	superiorfrontal_part9	4.5
lateraloccipital_part7	1.2	superiorfrontal_part10	5.2
lateraloccipital_part8	0.3	superiorfrontal_part11	4.8
lateraloccipital_part9	0.8	superiorfrontal_part12	6.1
lateralorbitofrontal_part1	3.3	superiorfrontal_part13	6.1
lateralorbitofrontal_part2	3.7	superiorparietal_part1	2.7
lateralorbitofrontal_part3	5.0	superiorparietal_part2	3.3
lateralorbitofrontal_part4	3.3	superiorparietal_part3	5.7
lateralorbitofrontal_part5	6.6	superiorparietal_part4	2.8
lingual_part1	2.5	superiorparietal_part5	0.7

lingual_part2	0.0	superiorparietal_part6	1.7
lingual_part3	2.1	superiorparietal_part7	5.4
lingual_part4	0.0	superiorparietal_part8	3.5
lingual_part5	1.4	superiorparietal_part9	9.1
lingual_part6	0.1	superiorparietal_part10	6.3
medialorbitofrontal_part1	2.7	superiortemporal_part1	6.9
medialorbitofrontal_part2	3.8	superiortemporal_part2	7.1
medialorbitofrontal_part3	2.3	superiortemporal_part3	6.2
medialorbitofrontal_part4	4.1	superiortemporal_part4	8.5
middletemporal_part1	4.1	superiortemporal_part5	9.4
middletemporal_part2	5.1	superiortemporal_part6	12.5
middletemporal_part3	6.5	superiortemporal_part7	13.0
middletemporal_part4	10.9	supramarginal_part1	9.4
middletemporal_part5	8.4	supramarginal_part2	6.0
middletemporal_part6	9.0	supramarginal_part3	5.9
parahippocampal_part1	7.1	supramarginal_part4	1.8
paracentral_part1	0.7	supramarginal_part5	3.2
paracentral_part2	3.4	supramarginal_part6	2.4
paracentral_part3	0.9	supramarginal_part7	2.9
parsopercularis_part1	3.6	frontalpole_part1	1.4
parsopercularis_part2	7.1	temporalpole_part1	4.5
parsopercularis_part3	7.1	transversetemporal_part1	12.5
parsorbitalis_part1	2.9	insula_part1	11.7
parstriangularis_part1	3.6	insula_part2	10.0
parstriangularis_part2	6.8	insula_part3	11.5
pericalcarine_part1	0.2	insula_part4	12.7
pericalcarine_part2	1.3		

Supplementary Table 1. **Tumor frequency percentage values at each parcel.**

**Glioma proto-oncogenes**	
IDH1	*TP53*
IDH2	*NF1*
TERT	*MDM2*
ATRX	*PIK3CA*
EGFR	*FUBP1*
CDKN2A	*NOTCH1*
CDKN2B	*PDGFRA1*
PTEN	*RIS*
RIK	*PI3K*

Supplementary Table 2. **List of glioma-related genes tested for enrichment among transcriptomic correlates of glioma distribution.** These genes were selected from a recent review of molecular genetic markers of adult glioma subtypes (Molinaro *et al.*, 2019).

**Brain Maps**	**Moran's I**
Tumor Frequency	0.69
Nodal Strength	0.30
OPC Distribution	0.29
PLS1 Loadings	0.49
PLS2 Loadings	0.54

Supplementary Table 3. **Spatial autocorrelation quantification via Moran's I for key brain map variables**.

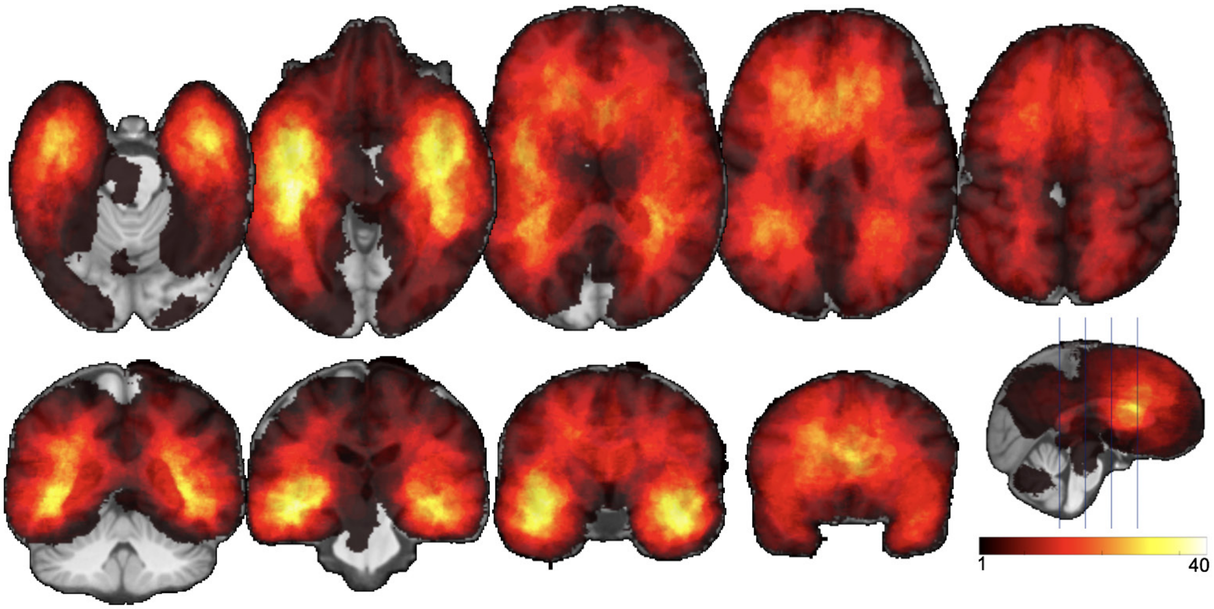

Supplementary Figure 1. **Raw lesion overlap map.** Colors indicate the number of lesions (out of a total of 335) overlapping with the associated voxel.

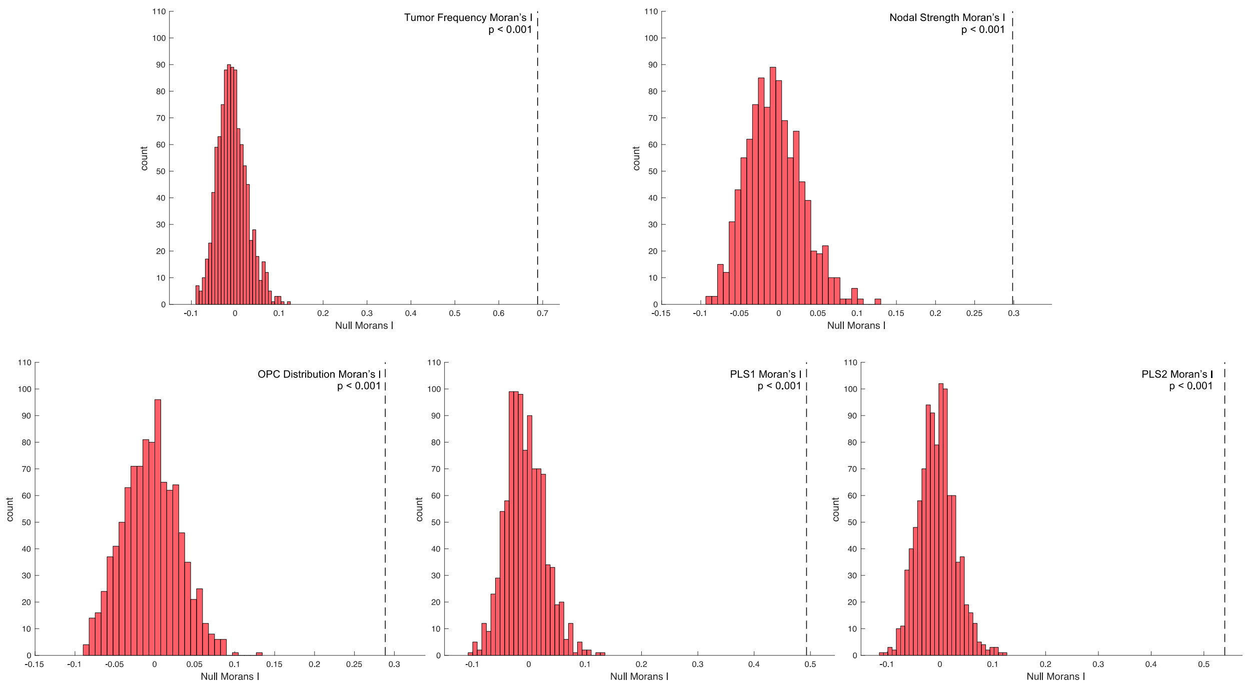

Supplementary Figure 2. **Permutation tests confirm non-random spatial autocorrelation structure of brain maps.** Moran's I for each brain map was compared to a distribution of Moran's I values for 1000 randomly permuted maps.

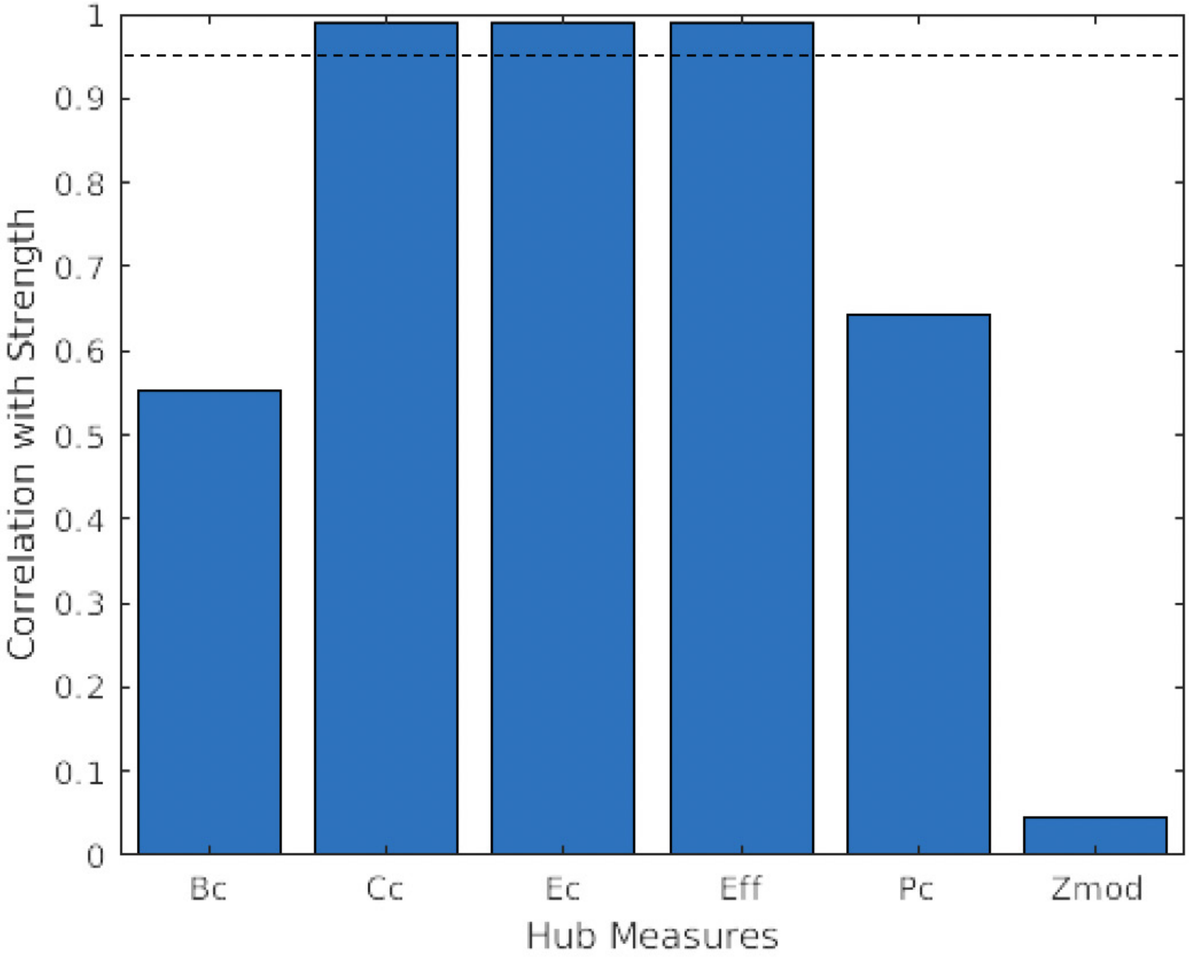

Supplementary Figure 3. **Rank correlations between nodal strength and other graph theoretical metrics of hubness.** Graph theoretical metrics with a correlation higher than the dotted line (rho=0.95) were screened from further analyses. While this threshold was arbitrary, the same measures would be screened across a variety of similar thresholds.

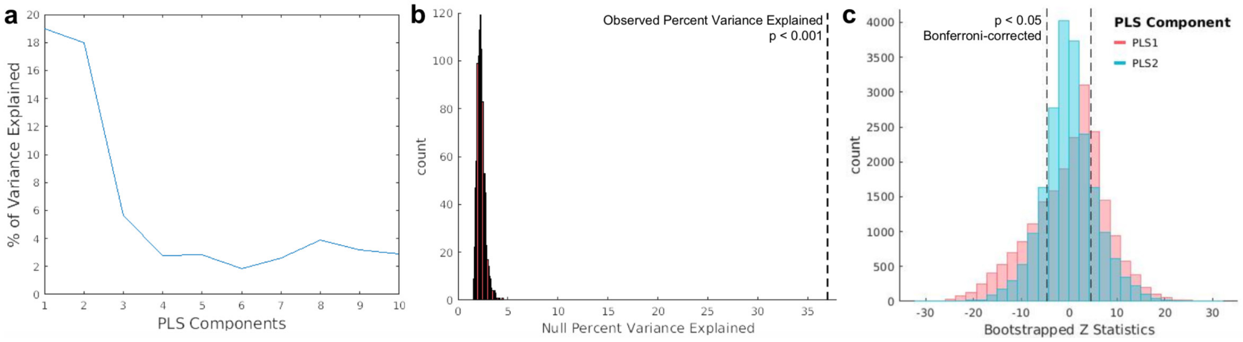

Supplementary Figure 4. **PLS regression analyses relating gene expression with glioma frequency.** A. Scree plot demonstrating percentage of variance explained by each subsequent PLS component. B. Percentage of variance explained across 1000 null models where the mapping of glioma frequency to gene expression is randomized, compared to the percent explained variance in the observed model, indicated by the dotted line. C. Distribution of bootstrapped Z statistics for each gene, corresponding to PLS1 and PLS2. Positive and negative Bonferroni-corrected significance thresholds are indicated by the dotted lines. These thresholds were not applied to the PLS gene lists.

References

Arloth J, Bader DM, Röh S, Altmann A. Re-Annotator: Annotation pipeline for microarray probe sequences. PLoS One 2015

Arnatkevičiūtė A, Fulcher BD, Fornito A. A practical guide to linking brain-wide gene expression and neuroimaging data. Neuroimage 2019

Fischl B. FreeSurfer. Neuroimage 2012

Hawrylycz MJ, Lein ES, Guillozet-Bongaarts AL, Shen EH, Ng L, Miller JA, et al. An anatomically comprehensive atlas of the adult human brain transcriptome. Nature 2012

Lake BB, Chen S, Sos BC, Fan J, Kaeser GE, Yung YC, et al. Integrative single-cell analysis of transcriptional and epigenetic states in the human adult brain. Nat. Biotechnol. 2018

Molinaro AM, Taylor JW, Wiencke JK, Wrensch MR. Genetic and molecular epidemiology of adult diffuse glioma [Internet]. Nat. Rev. Neurol. 2019; 15Available from: http://www.nature.com/articles/s41582-019-0220-2

Moran PAP. Notes on Continuous Stochastic Phenomena. Biometrika 1950

Richiardi J, Altmann A, Milazzo AC, Chang C, Chakravarty MM, Banaschewski T, et al. Correlated gene expression supports synchronous activity in brain networks. Science (80-.). 2015

Romero-Garcia R, Seidlitz J, Whitaker KJ, Morgan SE, Dolan RJ, Jones PB, et al. Schizotypy-related magnetization of cortex in healthy adolescence is co-located with expression of schizophrenia-related genes. Biol. Psychiatry 2019

Romero-Garcia R, Whitaker KJ, Váša F, Seidlitz J, Shinn M, Fonagy P, et al. Structural covariance networks are coupled to expression of genes enriched in supragranular layers of the human cortex. Neuroimage 2018
